# Prevalence of Self-Medication Practice and Associated Factors among Pregnant Women Who Attended Antenatal Care at Public Hospitals of North Shewa Zone, Amhara Region, Ethiopia

**DOI:** 10.1155/2024/6668480

**Published:** 2024-08-07

**Authors:** Abrham Demis, Birhanetensay Masresha Altaye, Mulugeta Emiru, Mitiku Tefera

**Affiliations:** ^1^ Department of Midwifery Debre Birhan Health Science College, Debre Birhan, Ethiopia; ^2^ Department of Pharmacy Asrat Woldeyes Health Science Campus Debre Birhan University, Debre Birhan, Ethiopia; ^3^ Department of Nursing School of Nursing and Midwifery Asrat Woldeyes Health Science Campus Debre Birhan University, Debre Birhan, Ethiopia

## Abstract

**Background:**

Self-medication practice is the use of medicine without consulting health professionals to treat self-recognized illness by the general population including pregnant women. Inappropriate self-medication practice during pregnancy may pose harmful consequences for the fetus as well as the mother. There is not given much attention on the practice of self-medication among pregnant women in our setting. Therefore, this study aimed to assess the prevalence of self-medication practice and associated factors among pregnant women who attended antenatal care at North Shewa Zone public hospitals.

**Methods:**

An institution-based cross-sectional study was conducted from June 01, 2022 to July 30, 2022, among 650 pregnant women who attended antenatal care at North Shewa Zone public hospitals. A multistage sampling technique was employed. The questionnaires were pretested. A structured interviewer-administered questionnaire and reviewed medical records were used for data collection. Epi-data version 4.6.2 and SPSS version 20 were utilized for data entry and analysis, respectively. Bivariate and multivariable logistic regression was done to identify associated factors, and *P* values less than 0.05 were considered statistically significant.

**Results:**

The prevalence of self-medication practice among pregnant women was 65.38%. Housewives (AOR = 0.097 95% CI 0.030, 0.310), farmers (AOR = 0.117, 95% CI 0.028, 0.493), people with health insurance (AOR = 0.507, 95% CI 0.300, 0.858), and people in preconception care (AOR = 0.038, 95% CI 0.011–0.135) were less likely to practice self-medication, while people with primary education (AOR = 3.00, 95% CI 1.217, 7.435), income less than 3,000 birr (AOR = 5.46, 95% CI 1.41, 21.1), participants in the first (AOR = 4.183, 95% CI 2.12, 8.24) and second trimesters (AOR = 2.05, 95% CI 1.18, 3.56), pregnant women who lived in rural areas (AOR = 1.579, 95% CI 1.103–2.260), and people who previously practiced self-medication (AOR = 8.2, 95% CI 5.04, 13.3) were more likely to practice self-medication.

**Conclusion:**

From the present finding, it can be concluded that self-medication among pregnant women is high. Previous self-medication practice, gestation period, educational status, monthly income, no preconception care, no health insurance, being a housewife, farmer, and place of residence were significantly associated with self-medication practice. Therefore, preventive measures such as proper counseling during dispensing, awareness creation programs on preconception care, and enrolling in health insurance programs to minimize the practice of self-medication are necessary.

## 1. Background

Self-medication is described as the selection and use of medicines, herbs, or home remedies based on an individual's own interests without a health professional's prescription to treat self-recognized illnesses and symptoms [1]. Inappropriate self-medication practice during pregnancy results in potential adverse effects on the fetus and increases the chance of drug resistance to the mother, treatment failure, misuse of medication, wastage of resources, and drug dependence [2]. Self-medication practice, however, may reduce health costs and save time spent waiting to see doctors for minor illnesses [3].

Self-medication practice (SMP) is currently a global problem [4]. Self-medication practice throughout pregnancy has been increasing in several regions of the world, particularly in developing countries [5, 6]. A study done by Shaikh showed that about 78% of drugs in developing countries were purchased without a prescription [7]. Despite the increment of self-medication among pregnant women all over the world, the majority are unaware of the consequences of self-medication [8].

In Ethiopia, self-medication during pregnancy is becoming more prevalent, and the reported prevalence ranges from 15.5% to 70% [9].

Different factors are implicated in self-medication practices. These include low socioeconomic status, prior experience with medications, use of drugs recommended by relatives, minor illness, lack of access to health services, uncontrolled distribution of drugs, patients' attitudes toward health care providers, long waiting times, drug costs, level of education, age, easy access to drugs without prescription, patient satisfaction, and beliefs about drugs and diseases [2, 10–14].

Rampant practice of self-medication during pregnancy may pose teratogenic and other harmful consequences to the fetus and mother, such as birth defects, miscarriage, allergic diseases, low birth weight, premature birth, developmental disorders, and fetal toxicity. It is estimated that about 10% of birth defects are caused by drug exposure in pregnant women [15]. The risk is higher when the drugs are used during the first trimester of pregnancy [16]. Self-medication can also worsen a patient's health and delay seeking medical advice from a health care provider [17].

Medicines used during pregnancy are usually based on an assessment of their harm to the mother and fetus. In most cases, the first choice of treatment for conditions during pregnancy is different from treatment for women who are not pregnant. Therefore, the choice of drug should be based on pregnancy risk categories which indicate the potential of a drug to cause birth defects if used during pregnancy [18].

Pregnancy-related drugs and medications have the potential to cross the placenta and affect the fetus. A variety of outcomes are possible, such as low birth weight, premature birth, miscarriage, stillbirth, and baby withdrawal after medications [19].

Worldwide, the practice of self-medication with over-the-counter (OTC) drugs among individuals is common. In countries like Ethiopia, even prescription drugs have been dispensed at a very high rate without a prescription which further increases risk of self-medication among pregnant women [20].

In addition to conventional self-medication practice, pregnant women may not realize that social drug use can affect the health of the fetus. About 3.6% of congenital anomalies are caused by substance use during pregnancy [21]. Regardless of the introduction of many maternal health care policy programs in Ethiopia such as free antenatal care and reduction exemption policy under the national health insurance system, antenatal visits for rural women are underutilized and have low attention on the risk of self-medication during pregnancy without prescription. Visits to antenatal care are declining due to low levels of education, lack of access to the media, distance from health centers, and unplanned pregnancies. Hence, this group favors self-medication and search for alternative medicine [22].

Many pregnant women have little awareness of the harmful impact of self-medication practice in Ethiopia, and there is no health policy to restrict self-medication practice during pregnancy in the country [15]. Even though some studies were conducted regarding self-medication practices among pregnant women in Ethiopia, still further studies are needed to get adequate data about the geographical area and population diversity of the country. The results of this study will serve as an input for the formulation of strategies or guidelines, and policies by program designers and policymakers to mitigate the potential risks of self-medication and its related interventions in antenatal care Therefore, the present study was carried out to show the prevalence of self-medication and associated factors among pregnant women in North Shewa Zone, Amhara region, Ethiopia 2022.

## 2. Method

### 2.1. Study Design, Area, and Period

An institution-based cross-sectional study was conducted among pregnant women who attended antenatal care at North Shewa Zone public hospitals from June 01, 2022, to July 30, 2022. The study was conducted in public hospitals of the North Shewa Zone. North Shewa Zone is one of the thirteen zones found in the Amhara regional state. Its city administration is Debre Berhan town, which is located 130 km from Addis Ababa and 695 km from Bahir Dar capital city of Amhara Regional state. Based on the Central Statistical Agency's 2007 population and housing census of North Shewa Zone, this Zone has a projected total population of 2,322,148 for the year 2019, of whom 1,171,510 are men and 1,150,638 are women [23]. The North Shewa Zone is bordered on the south and the west by the Oromia region, on the north by South Wollo, on the northeast by the Oromia Zone, and on the east by the Afar region. According to the Zonal Health Office Report, North Shewa has 164 private clinics, 97 governmental health centers, 391 health posts, 9 primary hospitals (of which two are private primary hospitals), 2 general hospitals, and one comprehensive specialized hospital. The North Shewa Zone has a total of 10 public and 2 private hospitals. The study was conducted from June 01, 2022, to July 30, 2022.

### 2.2. Population

#### 2.2.1. Source and Study Population

All pregnant women who attend antenatal care at North Shewa Zone public hospitals are the source population, and pregnant women who attended antenatal care at North Shewa Zone public hospitals during the study period are the study population.

#### 2.2.2. Eligibility Criteria

*(1) Inclusion Criteria*. All pregnant women who attended antenatal care in North Shewa Zone public hospitals within the study period were included.

*(2) Exclusion Criteria*. Pregnant women who were critically ill, unable to communicate at the time of data collection, and who came to return visits were excluded.

### 2.3. Sample Size Determination and Sampling Procedure

The sample size was determined based on a single population proportion formula using the study done in Kemissie is 26.9% [24], 5% margin of error at 95% confidence level, a design effect of 2 with a 10% nonresponse rate, and the calculated final sample size was 664.

A multistage sampling technique was employed to select study participants. There are 10 public hospitals in the North Shewa Zone; Debre Berhan Compressive Specialized Hospital (DBCSH), Enat Hospital (EH), Mehal Meda Hospital (MMH), Arerti Hospital (AH), Ataye Primary Hospital (APH), Shewarobit Hospital (SH), Deneba Hospital (DH), Debre Sina Hospital (DSH), Molalla Hospital(MH), and Midda Weremo Hospital (MWH). Among these, six were selected using a simple random sampling method. The selected hospitals are Debre Berhan Compressive Specialized Hospital (DBCSH), Enat Hospital (EH), Mehal Meda Hospital (MMH), Arerti Hospital (AH), Ataye Primary Hospital (APH), and Shewarobit Hospital (SH). The sample was proportionally allocated for each hospital, and the allocation was done based on previous 2 months' turnout for antenatal follow-up, which was 3014. The study participants were selected by the systematic random sampling technique. The sample interval value (*k*^th^) was 4, and the first participants were selected randomly by the lottery method from 1 to sample interval.

**Figure 1:**
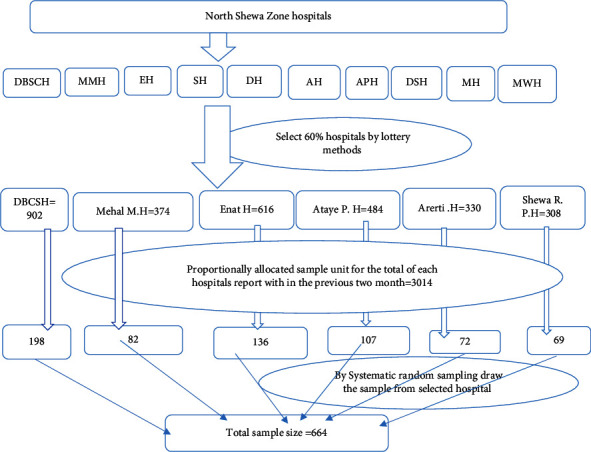
Schematic presentation of the sampling procedure for self-medication practice and its associated factors among pregnant women who attended antenatal care at public hospitals of North Shewa Zone, Amhara, Ethiopia, 2022.

The proportional allocation sampling technique was performed by (*nf* × *n*)/*N* (sample final *∗* number of total pregnant women attending antenatal care in each hospital/number of total pregnant women attending antenatal care within the previous 2 months) (Figure 1).

### 2.4. Operational Definitions and Definition of Terms

 
*Self-medication practice*: It is described as the use of medications (conventional or herbal) for self-identified illnesses or disorders without a prescription; it also includes the continuous use of medications for recurrent symptoms without a medical professional's order, either by buying them from an unauthorized body or sharing from other person [25].
 
*Over-the-counter medicine*: It refers to medicine that you can buy without a prescription.
 
*Emergency use*: It is required to meet the immediate needs of respondents.
 
*Preconception care*: It is a set of interventions that are to be provided before pregnancy [26].
 
*Better knowledge*: One should know the dose, indication, contraindication, and side effect of the drug.

### 2.5. Data Collection Procedure

A structured interviewer-administered questionnaire was prepared in English after a thorough literature review of previously validated published studies, and it contains five parts, namely, sociodemographic characteristics, obstetrics factors, conventional medicine practice, herbal medicine practice, and knowledge-related factors. The questionnaire was evaluated by an expert in the field. Then, the questionnaire was translated into Amharic language and translated back into English language to minimize translation errors. Finally, the Amharic version of a structured interviewer-administered questionnaire was applied to collect the data, such as sociodemographic characteristics, conventional medicine practice, herbal medicine practice, and knowledge-related factors, and reviewed patient medical records were used to extract the necessary obstetrics information. Six BSc midwives and six supervisors contributed to data collection. The supervisors gave the required information on issues of privacy, confidentiality, and consent seeking before the interview and starting with reviewing medical records. When collecting data, the data collectors used captured pictures of the drugs to easily remember what drugs were used.

### 2.6. Data Quality Assurance

In order to maintain the quality of the data, data collectors and supervisors received a one-day training session in data collection procedures. Before actual data collection time, the questionnaire was pretested on 5% (32) of the sample size at Deneba Hospital, and then possible adjustment or modification was made to the tool. The reliability of the knowledge section of the questionnaire was checked using Cronbach's alpha test, and the result was 70. Moreover, questionnaires were cross-checked daily for completeness, accuracy, and consistency immediately after data collection.

### 2.7. Data Analysis

The collected data were cleaned, coded, and entered into Epi-Data version 4.6.2 and then exported to SPSS version 20 for analysis. A multicollinearity test was carried out between the independent variable using variance inflation factors (VIF), and none was collinear (VIF: 1.02–2.5). Descriptive analysis was used to summarize the sociodemographic characteristics of respondents. The binary logistic regression model was performed for dichotomous variables. Bivariable logistic regression analysis was used to select candidate variables for multivariable logistic regression analysis. Variables with *P* value <0.25, in bivariable logistic regression, were entered into a multivariable logistic regression model to identify their independent association with the dependent variable. The regression model fitness was tested by Hosmer and Lemshow goodness test (Model 1 Sig. = 0.722, Model 2 Sig. = 0.143, and Model 3 Sig. = 0.943). Finally, results were presented using tables, graphs, figures, text, and charts. The strength of the association was reported as adjusted odds ratios, and statistical significance was set at *p* < 0.05 for all analyses.

### 2.8. Ethical Consideration

Ethical approval and clearance were obtained from Debre Berhan University, Asrat Woldeyes Health Sciences Campus Ethics Review Board with protocol number IRB-055 (Ref. No: IRB58/09/2014 EC). At all levels, officials were contacted and asked for permission. The purpose of the study was explained to the study participants, confidentiality was assured, and verbal consent was obtained before data collection.

## 3. Result

### 3.1. Sociodemographic Characteristics

A total of 650 pregnant women were recruited in this study with a response rate of 97.89%. More than one-third (32.3%) of the women was in the age group of 25–29 years. The mean age of the participants was 28.81 with a standard deviation of 5.8. Three hundred and forty-one (52.5%) mothers were living in rural areas. About two-thirds of the participants was Orthodox Christian by religion (65.7%). Most of the participants (88.8%) were attending primary school and above (88.8%), and married (84.1%). Two hundred and sixty-two (40.3%) pregnant women were housewives. The majority of respondents' (74.1%) income level was below 3,000 ETB. Half of them (50.6%) had valid health insurance to access free point of care from accredited health facilities (Table 1).

### 3.2. Obstetrics Characteristics of the Participants

More than half (54.2%) of the participants were in the second trimester. The majority of the pregnant women (74.4%) had a history of pregnancy, with 49.8% having two pregnancies. Seventy-six (16%) of them experienced stillbirth. Most of the participants (91.9%) had a history of previous ANC follow-up, and 14.3% of them reported previous pregnancy complications. The majority (82.9%) of pregnant women gave birth in governmental institutions (Table 2).

### 3.3. Self-Medication Practice during Pregnancy

About 65.38% of the pregnant women practiced self-medication. Of those who practiced self-medication, 30% (95% CL 26.5, 33.4) used conventional medicine and 45.2% (95% CI 41.2, 49) used herbal medicine, while 9.9% of pregnant women used both conventional and herbal medicines during their current pregnancy. A total of two hundred one and two hundred sixty-four women reported previous history of self-medication practice with conventional and herbal medicine, respectively (Table 3).

Hyoscine (9.5%), paracetamol (8.9%), aspirin (7.7%), amoxicillin (7.1%), and cough syrup (6.6%) were among the most widely used drugs.

Hyoscine (4.8%), aspirin (4.8%), amoxicillin (4%), ampicillin (3.8%), and tetracycline (1.6%) were more widely used drugs during the second trimester than the first and third trimesters of pregnancy, respectively (Table 4).

**Figure 2:**
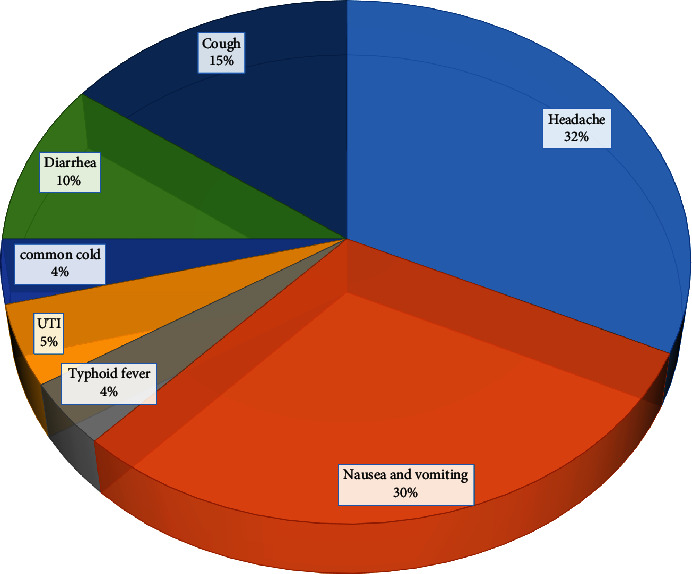
Indications of pregnant women who practice self-medication during pregnancy on conventional medicine at North Shewa Zone public hospitals, October 2022.

The participants' most commonly reported indications for self-medication with conventional medicine were headache (32%), nausea and vomiting (30%), and cough (15%) (Figure 2).

The most common source of conventional medicine was private and community pharmacies (26%), followed by friends (10.8%) and pregnant women themselves (8.3%).

Dama kesie (36.8%), tena dam (36.3%), and ginger (26.3%) were the widely used herbs for self-medication during pregnancy (Table 5).

### 3.4. Factors Associated with Self-Medication Practice during Pregnancy

A bivariate logistic regression analysis result showed that preconception care and previous self-medication on conventional and herbal medicine were factors with self-medication practice, whereas place of residence, distance from health facility, monthly income, alive child, and knowledge of pregnant women were considered for multivariable logistic regression analysis (Table 6). Pregnant women who have a previous history of self-medication on conventional medicine (AOR = 5.113, 95% CI 18, 8.23) and herbal medicine (AOR = 6.15, 95% CL 4.01, 9.43) were more likely to practice self-medication than who had not practiced self-medication previously. Pregnant women with preconception care (AOR = 0.47, 95% CL 0.30, 0.75) were less likely to practice self-medication than those without preconception care (Table 6).

Occupational status, monthly income, health insurance, preconception care, and previous self-medication were significantly associated factors with self-medication practice on conventional medicine. Educational status, gestational age, and gravidity were also considered for multivariable logistic regression analysis. Accordingly, occupational status, primary education, lower monthly income (<3,000 birrs), preconception care, first and second trimester pregnancy, health insurance, and previous self-medication practice were the significant associated factors with self-medication practice during pregnancy on conventional medicine (Table 7). In the present finding, housewives (AOR = 0.097 95% CI 0.030, 0.310) and farmer pregnant women (AOR = 0.117, 95% CI 0.028, 0.493) were less likely to practice self-medication on conventional medicine compared to those who were employed and students. Likewise, pregnant women with preconception care (AOR = 0.038, 95% CI 0.011–0.135) and health insurance (AOR = 0.507, 95% CI 0.300, 0.858) were less likely to practice self-medication as compared to those without preconception care and health insurance. On the other hand, participants with primary education (AOR = 3.00, 95% CI 1.217, 7.435), pregnant women with income less than 3,000 birr (AOR = 5.46, 95% CI 1.41, 21.1), participants in the first (AOR = 4.183, 95% CI 2.12, 8.24) and second trimesters (AOR = 2.05, 95% CI 1.18, 3.56), and participants with previous self-medication practice (AOR = 8.2, 95% CI 5.04, 13.3) were with high odds of practicing self-medication during their current pregnancy (Table 7).

Occupational status, educational status, residence, gestational age, and distance from the health facility were associated significantly with self-medication practice on herbal medicine during pregnancy in bivariate logistic regression analysis, and gravidity and monthly income were considered for multiple logistic regression analysis (Table 8).

Pregnant women who lived in rural areas (AOR = 1.94, 95% CI 1.34–2.80) and those who previously practiced self-medication (AOR = 9.76, 95% CI 6.73,14.16) were 1.94 and 9.76 times more likely to practice self-medication on herbal medicine as compared to those who lived in urban areas and who had not previously practiced self-medication (Table 8).

## 4. Discussion

This study investigated the prevalence of the practice of self-medication and associated factors among pregnant women who attended antenatal care in North Shewa Zone public hospitals. About 65.38% of pregnant women practice self-medication without a health professional's prescription. This study was comparable with studies conducted in the Jasika district of Ghana (68%) (47) and Kemisie Ethiopia (68.4%) [24]. The prevalence of self-medication practice in this finding was high when compared to reports in Nekemte Ethiopia (21.5%) (46), Iran (43.5%) [27], Brazil (36%) (42), Mexico (21.9%) (22), and Nepal (41.3%) [27–30]. On the contrary, it is lower than the finding in Ghana (74.1%) (27). The possible justification of this discrepancy might be differences over-the-counter medication pattern and prescription system of different counties of the world, especially in developing countries, including Ethiopia (48).

About 30% of the study participants practice self-medication on conventional medicine during their current pregnancy by themselves, because of the easy accessibility of medicine without prescription (14.1%) and prior experience with the drug (13.4%). These findings were almost comparable to studies in Harar (29.4%), Jimma (27.0%), Addis Ababa (26.6%), and Ethiopia (2, [10, 30]). The prevalence of self-medication practice on conventional medicine in the present study was higher when compared to reports in Bair Dar Ethiopia (25.1%), Bangladesh (12.2%), and Mexico (21.9%) (11, 24, 35). These study reports indicated that easy accessibility of medication without prescription and prior experience led to the practice ofself-medication among pregnant women. On, the contrary, it is lower than reports in Mwanza Tanzania (38.3%), Brazzaville bibintsene (46.24%), Ogbomoso oyoste Nigeria (51.0%), and Gedio Zone, Ethiopia (40.4%) [31–34]. The discrepancies in the findings could be due to the smaller sample sizes and different study periods and areas of the studies conducted in Mwanza, Tanzania; Brazzaville; Oyoste; and Gedio, Ethiopia.

In the present study, 45.2% of the participants were to practice self-medication on herbal medicine during their current pregnancy. The findings of this study were in line with studies done in Mwaniz Tanzania (46.24%) (8) and Kemisie Ethiopia (48.9%) [24]. The prevalence of the practice of self-medication on herbal medicine in the present study was higher as compared to studies in Harar Ethiopia (40.6%) [2], Mwanza Tanzania (37.5%) [35], and Uganda (20%) [36]. On the contrary, it is lower than reports in Iran (57.1%) [37] and Hosanna, Ethiopia (73.1%) [38]. This discrepancy could be due to the difference in the health-seeking behavior across the study setting, easy accessibility of herbal medicines, socio-cultural differences, and perception of herbal medicine in the study population.

Paracetamol (8.9%), aspirin (7.7%), tetracycline (3.4%), amoxicillin (7.1%), hyoscine (9.5%), ampicillin (6.6%), cimetidine (0.5%), panadol (0.5%), diclofenac (0.5%), pyridoxine (0.3%), promethazine (0.3%), ciprofloxacin (0.2%), metoclopramide (0.2%), and omeprazole (0.2%) were among commonly used medications for self-medication during pregnancy without prescription. This suggested that some pregnant women in this study were potentially at higher risk. To illustrate, tetracycline has known teratogenic effects such as permanent teeth discoloration and impairment of fetal long bone growth. The use of aspirin in the first trimester of pregnancy causes an increased risk of overall congenital malformation [19].

Common herbal medicine used by pregnant women were ginger (Zingier officinal) (26.3%), garlic (11.7%), tenadam (Ruta chalepensis) (36.3%), damakesie (Ocimum lamiifolium) (36.8%), and thymes (tosign) (5.7%). This study is supported by studies conducted in Jimma Ethiopia, Bukavo, Eastern DR Congo, and Kemisie Ethiopia [8, 14, 24]. This herbal medicine is the most prominent and widely practiced by a wider population and pregnant women around the world because of the healing benefits. But, its harmful effect is not known. Because many herbal medicines are poorly studied, it is not possible to rule out their teratogenicity effects [39].

The findings of this study revealed that occupational status was significantly associated with self-medication practice. This study is supported by studies conducted in Mwanza Tanzania, Ghana, and Nekemte Ethiopia [33, 40, 41]. Pregnant women who were housewives 91% and farmers 88.3% were less likely to practice self-medication than pregnant women who were employed and students. Due to the expansion of medical information, time constraints, and media exposure to pharmaceutical promotion, both employed individuals and students have risk to practice self-medication than housewives and farmers [42].

Based on this, the educational status of pregnant women with primary education was significantly associated with self-medication. This is similar to a study conducted in Bahir Dar, Ethiopia, Jasika Ghana, and Lira Uganda [10, 43, 44]. The odds of self-medication practice among women whose educational level is primary school were 3.0 times the odds of those with diploma or degree. One explanation for the factors could be that women in diploma or degree programs are more aware of the risks associated with the drug used during pregnancy than those with primary-level education. So, at the diploma or degree level, there is less drug use without a prescription during pregnancy [45].

Participants who had low monthly income (<3,000 birrs) were 5.46 times more likely to use self-medication compared to those who had high monthly income (>6,000 birrs). The current result is consistent with studies done in Harar Ethiopia, Bahir Dar Ethiopia, Iran, Goba Ethiopia, and Bukavu Eastern DR Congo [2, 10, 11, 13, 14]. It might be justified that low-income pregnant women may not be able to afford medical facilities and consult licensed medical professionals. Therefore, they might be urged to purchase cheaper drugs from over-the-counter centers without a prescription, which in turn leads to high-level self-medication practices.

Pregnant women who had valid health insurance were 49.3% less likely to practice self-medication than those who had no valid health insurance. This study is congruent with studies in Northern Ghana (40) and Tigray Ethiopia [46]. Valid health insurance helps to pay for health care services, and it can help cover services ranging from routine health care visits to major medical costs for a serious illness. As a result, pregnant women are more likely to follow their prescription because their insurance will pay for both their appointment and their medication. Therefore, health providers help pregnant mothers to enroll for health insurance.

Pregnant women who had preconception care were nearly 53–77.4% less likely to utilize self-medication as compared to those who had not had preconception care before pregnancy, because pregnant women might take advice before conception or early in pregnancy to maximize the health outcome of pregnancy. Health professionals create awareness on the harmful effect of self-medication without prescription.

The finding of this study revealed that the first and second trimesters of gestational age were significantly associated with self-medication practice. This is similar to a study conducted in Addis Ababa Ethiopia, Northern Jordan, and Ibadan Nigeria [47–49]. The odds of self-medication practice among women in their first and second trimesters of pregnancy were 4.18 and 2.05 times the odds of those in their third trimester of pregnancy. Because women often have greater symptoms and discomfort during the first trimester of pregnancy, they frequently need to take their medications. However, this is the more critical time concerning fetal damage associated with drug use. Therefore, more emphasis should be given to the use of medication in the first and second trimesters of pregnancy [50].

A previous history of self-medication was 5.11–8.2 times more likely to lead to self-medication as compared to not having previous experience of the same. These factors are similar to the study findings in Addis Ababa Ethiopia and Lira Uganda [43, 48]. A possible explanation for this might be that pregnant women would have used self-medicine before they became aware of disease and treatment options, which encourages them to utilize self-medication rather than an alternative course of action.

Pregnant women who lived in rural areas and had previously practiced self-medication with herbal medicine were 1.94 times and 9.76 times more likely to practice self-medication on herbal medicine as compared to those who lived in the urban area and who had not practiced self-medication previously. These factors are similar to studies in Mwanza, Tanzania [35]. A possible justification might be that pregnant women who lived in a rural community have low access to antenatal care counseling about the risk of herbal medicine and are familiar with herbal medicine to treat minor illnesses [51].

## 5. Conclusion and Recommendation

According to the study's findings, self-medication by pregnant women is found to be high. It is a huge problem and has to be reduced or prevented with the use of efficient measures and interventions. Factors like having no valid health insurance, no preconception care, low monthly income, primary school educational status, and previous experience of self-medication practice were the predictors of self-medication practice on conventional medicine. Place of residence and previous self-medication with herbal medicine were the factors associated with self-medication with herbal medicine during their current pregnancy. The high magnitude and the associated factors require great attention, especially for pregnant women. Considerable attention should be given to increasing pregnant women's access to maternal care services by enrolling them in valid health insurance, lowering the cost of maternal care services, avoiding the provision of medications without a medical prescription, creating a culture using the public media to prevent self-medication, and creating awareness about preconception care. Finally, the health professionals educating the general population and enhancing pregnant women's awareness about the potential risks of self-medication during pregnancy at the antenatal care level are crucial. Moreover, the health provider should emphasize more on the implementation of preconception care and national health insurance to reduce the risk of self-medication without prescription during pregnancy.

### 5.1. Limitation

The limitations of this study may be recall bias which possibly underestimates self-medication practice. This study is quantitative; it would be preferable if a qualitative method was used to analyze study subjects' perspectives toward self-medication.

## Figures and Tables

**Table 1:** Sociodemographic characteristics of pregnant women who attended antenatal care at North Shewa Zone public hospitals, October 2022.

Variables	Frequency	Percentage (%)
*Age of participant (year)*		
18-19	44	6.7
20–24	119	18.3
25–29	210	32.3
30–34	155	23.9
35–39	93	14.3
40–49	29	4.5

*Monthly income*		
<3,000 birr	482	74.1
3,000–6,000 birr	139	21.4
>6,000 birr	29	4.5

*Marital status*		
Single	37	5.7
Married	547	84.1
Divorced	53	8.2
Windowed	13	2

*Occupational status*		
Government employed	141	21.7
Private employed	172	26.5
Housewife	262	40.3
Farmer	52	8.0
Student	23	3.5

*Educational status*		
Unable to read and write	73	11.2
Primary school	148	22.8
Secondary school	232	35.7
College or university student	57	8.8
Diploma or degree	140	21.5

*Religious*		
Orthodox	152	65.7
Muslim	71	23.4
Protestant	427	10.9

*Residence*		
Urban	309	47.5
Rural	341	52.5

*Distance from health facility*		
<5 km	348	54
6–10 km	126	19.5
>10 km	171	26.5

*Health insurance*		
Yes	329	50.6
No	321	49.4

**Table 2:** Obstetric characteristics of pregnant women who attended antenatal care at North Shewa Zone public hospitals, October 2022.

Variable	Frequency	Percentage (%)
*Gestational age*		
First trimester	116	17.8
Second trimester	352	54.2
Third trimester	182	28.0

*Gravidity*		
First pregnancy	166	25.6
Second pregnancy	324	49.8
Third and above pregnancy	160	24.6

*Stillbirth*		
Yes	76	16
No	408	84

*Previous antenatal follow-up*		
Yes	445	91.9
No	39	8.1

*Previous pregnancy complication*		
Yes	69	14.3
No	415	85.7

*If yes*		
Vaginal bleeding	29 (42)	42
Premature rupture of membrane	9 (13)	13
Severe headache	5 (7.2)	7.2
Blurring of vision	12 (17.4)	17.4
Edema	5 (7.2)	7.2
Loss of fetal movement	4 (6)	6
Preterm labor	2 (2.9)	2.9
Abdominal pain	3 (4.3)	4.3

*Preconception care*		
Yes	138 (21.2)	21.2
No	512 (78.8)	78.8

**Table 3:** .Frequency distribution of self-medication practice before and during pregnancy on conventional and herbal medicine at North Shewa Zone public hospitals, October 2022.

Variable	Frequency	Percentage (%)
*SMP on modern medicine during their current pregnancy*		
Yes	195	30
No	455	70

*Self-medication practice on modern medicine previously*		
Yes	201	30.9
No	449	69.1

*SMP on herbal medicine during their current pregnancy*		
Yes	294	45.2
No	356	54.8

*Self-medication practice on herbal medicine previously*		
Yes	264	40.6
No	386	59.4

**Table 4:** Drugs commonly practiced at each trimester of pregnancy.

Drugs commonly practiced	First trimester of pregnancy	Second trimester of pregnancy	Third trimester of pregnancy
Amoxicillin	14 (2.3%)	24 (4%)	8 (1.3%)
Paracetamol	28 (4.6%)	20 (3.3%)	10 (1.6%)
Aspirin	14 (2.3%)	29 (4.8%)	7 (1.1%)
Ampicillin	13 (2.1%)	23 (3.8%)	8 (1.3%)
Tetracycline	6 (1%)	10 (1.6%)	6 (1%)
Hyoscine	18 (3%)	29 (4.8%)	15 (2.5%)
Cimetidine	1 (0.2%)	0	2 (0.3%)
Ciprofloxacin	1 (0.2%)	0	0
Diclofenac	0	2 (0.3%)	1 (0.2%)
Metoclopramide	0	1 (0.2%)	0
Omeprazole	0	0	1 (0.2%)
Panadol	0	1 (0.2%)	0
Pyridoxine	0	2 (0.3%)	0
Promethazine	1 (0.2%)	1 (0.2%)	0

**Table 5:** Types of herbal medicine and source of information about herbal medicine for pregnant women who practice self-medication during their pregnancy.

Variable	Frequency	Percentage (%)
Ginger (Zingiber officinale)	171	26.3
Garlic	76	11.7
Tenadam (*Ruta chalepensis*)	236	36.3
Damakesie (*Ocimum lamiifolium*)	239	36.8
Thymes (tosign)	37	5.7

**Table 6:** Model-1. Bivariate and multivariable logistic regression analyses of variables' significant association with self-medication practice during pregnancy in North Shewa public hospitals, October 2022 (*n* = 650).

Variable	Self-medication practice		COR (95% CI)	AOR (95% CI)	*P* value
	Yes	No			
*Residence*					
Rural	247	94	1.93 (1.39, 2.68)	1.93 (1.39, 2.68)	0.151
Urban	178	131	1		

*Monthly income*					
3,000	332	150	3.62 (1.66, 7.85)	1.86 (0.75, 4.63)	0.179
3,000–6,000	82	57	2.35 (1.03, 5.36)	1.90 (0.76, 4.77)	1.90 (0.76, 4.77)
>6,000	11	18	1		

*Distance from health facility*					
>10 km	128	43	2.02 (1.35, 3.04)	1.06 (0.56, 2.02)	1.06 (0.56, 2.02)
5–10 km	86	40	1.46 (0.95, 2.25)	0.87 (0.46, 1.66)	0.87 (0.46, 1.66)
5 km	207	114	1		

*Preconception care*					
Yes	71	68	0.46 (0.31, 0.67)	0.47 (0.30, 0.75)	0.002^*∗*^
No	354	157	1		

*Previous self-medication on conventional medicine*					
Yes	172	29	4.59 (2.97, 7.1)	5.11 (3.18, 8.23)	0.001^*∗*^
No	253	196	1		

*Previous self-medication on herbal medicine*					
Yes	222	342	4.76 (3.24, 7.0)	6.15 (4.01, 9.43)	0.001
No	203	183	1		

*Knowledge of patient*					
Good knowledge	392	190	0.45 (0.27, 0.70)	0.68 (0.37, 1.24)	0.211
Poor knowledge	33	35	1		

*Notes*. 1 = reference; ^*∗*^*P* < 0.05.

**Table 7:** Model-2. Bivariate and multivariable logistic regression analyses of variables' significant association with self-medication practice on conventional medicine during pregnancy in North Shewa Zone public hospitals, October 2022 (*n* = 650).

Variables	Self-medication practice		COR (95% CI)	AOR (95% CI)	*P* value
	Yes	No			
*Occupational status*					
Government employed	52	89	0.449 (0.184–1.097)	1.847 (0.470–7.258)	0.380
Private employed	87	85	0.787 (0.328–1.892)	0.647 (0.214–1.957)	0.441
Housewife	35	227	0.119 (0.048–0.291)	0.097 (0.030–0.310)	0.001^*∗*^
Farmer	8	44	0.140 (0.046–0.427) 1	0.117 (0.028–0.493)	0.003^*∗*^
Student	13	10	1	1	

*Educational status*					
Unable to read and write	16	57	0.678 (0.349–1.316)	2.248 (0.780–6.483)	0.134
Primary school	47	101	1.124 (00.680–1.857)	3.00 (1.217–7.435)	0.017^*∗*^
Secondary school	78	154	1.223 (0.776–1.927)	1.847 (0.820–4.161)	0.139
College and university	13	44	0.713 (0.348–1.463)	0.761 (0.301–1.927)	0.565
Diploma or degree	41	99	1	1	

*Monthly income*					
<3,000 birr	158	323	3.077 (1.053–8.991)	5.46 (1.41–21.1)	0.014^*∗*^
3,000–6,000 birr	32	107	1.869 (0.606–5.769)	1.25 (0.361–4.368)	0.721
>6,000 birr	4	25	1	1	

*Health insurance*					
Yes	86	243	0.688 (0.491–0.965)	0.507 (0.300–0.858)	0.011^*∗*^
No	109	212	1	1	

*Preconception care*					
Yes	10	129	0.137 (0.07–0.266)	0.126 (0.056–0.284)	0.001^*∗*^
No	185	326	1	1	

*Pregnancy trimesters*					
First	52	64	2.884 (1.737–4.789)	4.183 (2.12–8.24)	0.001^*∗*^
Second	103	249	1.468 (0.966–2.233)	2.05 (1.18–3.56)	0.011^*∗*^
Third	40	142	1	1	

*Gravidity*					
First	52	114	1.324 (0.817–2.147)	1.268 (0.634–2.538)	0.502
Second	102	222	1.334 (0.872–2.041)	1.54 (0.94–2.998)	0.080
Third and above	41	119	1	1	

*Previous SMP on conventional medicine*					
Yes	118	83	6.868 (4.731–9.972)	8.2 (5.04–13.3)	0.001
No	77	372	1	1	

*Notes*. 1 = reference; ^*∗*^*P* value <0.05.

**Table 8:** Model-3. Bivariate and multivariable logistic regression analyses of variables significant association with self-medication practice on herbal medicine during pregnancy in North Shewa Zone public hospitals, October 2022 (*n* = 650).

Variables	SMP		COR (95% CI)	AOR (95% CI)	*P* value
	Yes	No			
*Occupational status*					
Government employed	55	86	1	1	
Private employed	67	105	0.998 (0.632–1.507)	0.862 (0.531–1.399)	0.547
Housewife	137	125	1.714 (1.130–2.599)	1.424 (0.898–2.258)	0.133
Farmer	29	23	1.972 (1.036–3.752)	0.354 (0.663–2.766)	0.405
Student	6	17	0.552 (0.205–1.486)	0.503 (0.185–1.371)	0.179

*Educational status*					
Unable to read and write	39	34	1.941 (1.094–3.445)	1.184 (0.487–3.167)	0.664
Primary school	72	76	1.603 (1.001–2.567)	1.055 (0.533–2.086)	0.878
Secondary school	111	121	1.552 (1.011–2.384)	1.240 (0.677–2.271)	0.486
College and university	20	37	0.915 (0.481–1.740)	1.068 (0.540–2.115)	0.850
Diploma or degree	52	88	1	1	

*Monthly income*					
<3,000 birr	226	256	1.962 (0.876–4.396)	1.243 (0.487–3.167)	0.649
3,000–6,000 birr	59	80	1.639 (0.697–3.856)	1.441 (0.595–3.486)	0.418
>6,000 birr	9	20	1	1	

*Health insurance*					
Yes	166	163	1.536 (1.125–2.095)	1.025 (0.675–1.556) 1	0.0.909
No	128	193	1		

*Distance from health facility*					
<5 km	138	210	1	1	0.898
5−10 km	62	64	1.474 (0.978–2.221)	1.037 (0.593–1.815)	0.592
>10 km	91	80	1.731 (1.197–2.504)	1.160 (0.673–2.000)	

*Pregnancy trimesters*					
First	50	66	0.725 (0.454–1.158)	0.833 (0.506–1.374)	0.475
Second	151	201	0.719 (0.502–1.030)	0.752 (0.514–1.099)	0.131
Third	93	89	1	1	

*Gravidity*					
First	70	96	1	1	0.440
Second	143	181	1.084 (0.742–1.581)	0.846 (0.554–1.292)	0.952
Third and above	81	79	1.406 (0.908–2.177)	0.985 (0.601–1.613)	

*Previous SMP on herbal medicine*					
Yes	200	64	9.70 (6.73, 13.98)	9.76 (6.73, 14.16)	0.001^*∗*^
No	94	292	1	1	

*Residence*					
Rural	177	164	1.771 (1.295–2.422)	1.94 (1.34–2.80)	0.001^*∗*^
Urban	117	192	1	1	

*Notes*. 1 = reference; ^*∗*^*p* < 0.05.

## Data Availability

The raw data used to support the findings of this study are made available from the corresponding author upon reasonable request.

## Abbreviations

ANC: Antenatal care
AOR: Adjusted odds ratio
DBU: Debre Berhan University
CI: Confidence interval
COR: Crude odd ratio
MMR: Maternal mortality rate
IRB: Institutional Review Board
OTC: Over-the-counter
SDG: Sustainable development goal
SMP: Self-medication practice
USAID: United States Agency for International Development
WHO: World Health Organization.
